# Puberty Starts in the Gut: Intestinal Clues to Early Puberty-Rethinking Biomarkers in Pediatric Endocrinology

**DOI:** 10.3390/ph19010049

**Published:** 2025-12-25

**Authors:** Otilia Elena Frăsinariu, Teodora Cristina Vintilă, Ioana Vasiliu, Violeta Ștreangă, Aniela Rugină, Oana Raluca Temneanu, Ionuț Daniel Iancu, Andreea Iațentiuc, Elena Jechel, Alexandru Florescu

**Affiliations:** 1 Grigore T. Popa University of Medicine and Pharmacy, 700115 Iași, Romania; frasinariu.otilia@umfiasi.ro (O.E.F.); ioana_medgen@yahoo.com (I.V.); violeta.streanga@umfiasi.ro (V.Ș.); aniela.rugina@umfiasi.ro (A.R.); temneanu.oana@umfiasi.ro (O.R.T.); iancu.ionut-daniel@email.umfiasi.ro (I.D.I.); andreeacimpan@yahoo.com (A.I.); elena.jechel@yahoo.com (E.J.); alexandru-florin.florescu@umfiasi.ro (A.F.); 2 Endocrinology Department, Saint Spiridon County Hospital, 700111 Iași, Romania

**Keywords:** gut microbiota, dysbiosis, HPG axis, precocious puberty, short-chain fatty acids, estrobolome

## Abstract

Central precocious puberty (CPP) may be influenced by gut microbiota through changes in short-chain fatty acids (SCFAs), β-glucuronidase activity, and enterohepatic estrogen recycling. This narrative review integrates current evidence from human and animal studies exploring microbial contributions to pubertal timing. Across multiple cohorts, CPP is associated with loss of SCFA-producing commensals, such as *Bacteroides*, and increased abundance of taxa like *Alistipes*, *Ruminococcus*, and *Lachnoclostridium*. These microbial shifts are linked to altered SCFA profiles, diminished anti-inflammatory and neuroendocrine modulation, and enhanced reabsorption of estrogens via microbial β-glucuronidase activity. Experimental models support a causal connection: gut dysbiosis accelerates pubertal onset, whereas microbiota-targeted interventions can restore hormonal balance and delay activation of the HPG axis. While some overlap with obesity-associated microbiota exists, the endocrine-specific microbial changes observed in CPP suggest partially distinct mechanisms. Overall, the gut microbiota emerges as both a modulator and potential biomarker of early pubertal onset. Its integration into pediatric endocrine frameworks could improve early risk assessment and guide future interventions, though further validation through standardized, longitudinal, and diverse population studies is still required.

## 1. Introduction

The timing of pubertal onset is a tightly regulated process governed by the hypothalamic–pituitary–gonadal (HPG) axis, traditionally viewed through the lens of neuroendocrine and genetic regulation [1,2]. However, in recent years, a growing body of research has begun to challenge this classical paradigm, suggesting that signals arising from the gastrointestinal tract, particularly those mediated by the gut microbiota, may play a critical role in shaping endocrine transitions [3]. Among these, the possibility that gut microbial components or metabolites could serve as early, non-invasive biomarkers of pubertal onset or dysregulation is gaining increasing attention in pediatric endocrinology [3,4].

The concept of the gut–brain–gonad axis introduces a new layer of complexity to our understanding of pubertal regulation. Gut microorganisms and their byproducts, such as short-chain fatty acids (SCFAs), inflammatory mediators, and estrogen-modifying enzymes (e.g., β-glucuronidase) have been implicated in modulating key processes related to sexual maturation [3,5]. Notably, these microbial signals may influence the timing and progression of puberty through pathways involving systemic inflammation, energy metabolism, and direct neuroendocrine interactions at the level of the hypothalamus [6,7].

Central precocious puberty (CPP), defined as the premature activation of the HPG axis, presents an ideal clinical model for exploring these gut-derived influences [8]. Emerging evidence suggests that children with CPP exhibit distinct alterations in gut microbial composition compared to age-matched peers [8,9]. These changes, often linked to obesity, dietary patterns, antibiotic exposure, and immune activation, raise the question of whether specific microbial profiles or functional signatures could serve as predictive or diagnostic biomarkers of early puberty [9,10,11].

This review aims to critically evaluate current evidence linking the gut microbiota to pubertal development, with a specific focus on its potential as a biomarker platform for early pubertal onset. We explore mechanistic pathways through which microbial taxa, enzymes, and metabolites may interact with the HPG axis, highlight early-life factors that shape microbial architecture and pubertal timing and assess the strengths and limitations of current human and animal studies. Finally, we outline key research gaps and propose future directions for integrating microbiome-based diagnostics into clinical pediatric endocrinology.

Given the heterogeneity of available evidence, it was essential to outline the approach used in selecting the literature for this narrative review. To contextualize the evidence synthesized, we performed a broad, nonsystematic search of the literature examining the relationship between gut microbiota and pubertal development. The search was conducted across major biomedical databases, including PubMed, Scopus, Web of Science, and Google Scholar, and focused on studies published between January 2020 and September 2025. This interval was selected because research on the gut microbiota–endocrine interface expanded substantially after 2020, driven by advances in sequencing technologies, microbial functional profiling, and improved analytical standards. Earlier influential studies were also considered when identified through the reference lists of key publications. We prioritized research that investigated gut microbial composition or functional activity in relation to central precocious puberty or early pubertal onset, in either human cohorts or relevant animal models, and that provided quantitative or mechanistic insights involving endocrine markers such as LH, FSH, or estradiol. Studies focusing exclusively on male puberty, unrelated endocrine disorders, or metabolic conditions without pubertal assessment were not considered central to the aims of this synthesis, nor were those lacking microbiota profiling or functional microbial analyses. Literature retrieval relied on combinations of terms such as “gut microbiota,” “intestinal microbiome,” “intestinal flora,” “precocious puberty,” “early puberty,” “pubertal timing,” “girls,” and “children,” ensuring coverage of both compositional and functional dimensions. No language-based restrictions were applied in order to minimize selection bias and to obtain a comprehensive and globally representative overview of current research in this evolving field.

## 2. Neuroendocrine Modulators of Pubertal Timing: A Framework for Microbiota Interactions

The initiation of puberty is orchestrated by the activation of the hypothalamic–pituitary–gonadal axis, triggered by increased pulsatility of gonadotropin-releasing hormone (GnRH) secretion from specialized hypothalamic neurons. This, in turn, stimulates the release of luteinizing hormone (LH) and follicle-stimulating hormone (FSH), promoting gonadal maturation and sex steroid production [12,13,14]. While the sequence of events is well defined, the upstream triggers of GnRH pulsatility remain incompletely understood.

Among the most critical regulators is the kisspeptin system, which serves as a central integrator of metabolic and hormonal signals. Peripheral cues such as leptin, insulin, IGF-1, ghrelin, and cortisol modulate kisspeptin neurons, thereby linking nutritional and stress-related states to pubertal onset [15,16,17]. In particular, leptin, a hormone secreted by adipose tissue, acts permissively by signaling adequate energy reserves to the hypothalamus [18,19]. Leptin deficiency results in pubertal failure, while hyperleptinemia, commonly observed in childhood obesity, is associated with earlier pubertal onset, albeit modulated by leptin resistance and inflammation [20].

Systemic low-grade inflammation, often driven by excess adiposity or metabolic stress, further influences pubertal timing by disrupting leptin signaling and impairing GnRH regulation [21,22]. Cytokines such as IL-6 and TNF-α have been shown to interfere with hypothalamic function and reproductive hormone secretion [23,24].

These neurometabolic and immune pathways are increasingly recognized as key points of interaction with the gut microbiota. Gut-derived signals including SCFAs, cytokine modulators, and estrogen-metabolizing enzymes may influence pubertal development through their effects on metabolic homeostasis, inflammatory tone, and neuroendocrine signaling [25,26].

## 3. Precocious Puberty in Girls: The Clinical Relevance of Early Biomarkers

Central precocious puberty is defined by the premature activation of the HPG axis, with the onset of secondary sexual characteristics before the age of 8 in girls. Although the endocrine cascade remains intact, its early initiation has profound implications, including accelerated bone maturation, compromised adult height, and psychosocial disorders [27,28]. It not only affects their adult height and psychological well-being but also increases the risk of various diseases in the adulthood including hypertension, stroke, ischemic heart disease, type 2 diabetes and estrogen dependent cancer [29,30,31,32].

The global rise in CPP incidence, particularly in urbanized populations, has prompted investigations into modifiable environmental and biological contributors [33,34,35]. While known factors include obesity, endocrine-disrupting chemicals (EDCs), and psychosocial stress, increasing attention is being directed toward the gut microbiota as both a modulator and potential early biomarker of pubertal timing [36,37,38,39,40].

Microbial signatures associated with CPP may reflect underlying alterations in inflammation, estrogen metabolism, or energy signaling all of which intersect with established neuroendocrine regulators. Thus, profiling the gut microbiota offers a promising, non-invasive approach to identifying children at risk of early pubertal onset and to guiding personalized interventions [41,42,43].

Idiopathic central precocious puberty (ICPP) denotes GnRH-dependent early activation of the HPG axis without an identifiable CNS lesion. It accounts for most CPP cases in girls and is characterized by progressive thelarche, growth-spurt, and advanced bone age [44]. Diagnosis rests on a pubertal-range LH (basal or after a GnRH/GnRH-agonist stimulation test), supportive estradiol levels, and, when used, pelvic ultrasound showing increased uterine/ovarian volume with follicles [30,45,46]. The GnRH stimulation test represents the gold standard test for differentiation between CPP and peripheral precocious puberty. If the LH peak (after 120 min after the GnRH analog injection) is higher than 5 IU/L and the FSH/LH ratio is higher than 0,6 a diagnosis of CPP is made [30,47]. Given the discomfort associated with the test, there is a pressing need to advance non-invasive strategies. Stool-based candidates, including gut microbiota composition changes and related profiles, offer a promising direction.

Standard treatment is long-acting GnRH analogs (leuprolide, triptorelin), which suppress gonadotropins and improve predicted adult height while easing psychosocial strain [48,49]. Although effective, standard approaches involve frequent injections that can be painful and distressing, undermining compliance, increasing costs, and potential adverse reactions including transient vaginal bleeding, allergic reactions, and local sterile abscesses that may reduce treatment effectiveness [50]. Optimizing existing strategies for CPP is therefore urgent to enhance acceptability in children. Emerging stool-based interventions, particularly probiotics and fecal microbiota transplantation, warrant rigorous evaluation as less invasive options. Ultimately, the gut microbiome’s influence on endocrine signaling highlights its relevance to future therapy design [51].

Against this background, early, non-invasive biomarkers (such as gut-microbiota–based signatures) are clinically appealing to flag at-risk girls before unequivocal signs, prioritize referral, and avoid unnecessary imaging or delayed therapy [38].

## 4. Development and Maturation of the Gut Microbiota: Evolution Across the Lifespan

The human gut microbiota undergoes substantial changes throughout life, influenced by host genetics, diet, environment, immune function, and hormonal status. Understanding the developmental trajectory of the gut microbiota is critical to elucidating its potential role in regulating systemic processes such as immunity, metabolism, and endocrine function including its emerging association with pubertal timing [52,53].

The first 1000 days of life, spanning gestation through the second year, are now recognized as a critical window for microbial programming of host physiology. During this period, the gut microbiota interacts intimately with the developing immune system, helping establish tolerance to self and environmental antigens while educating mucosal and systemic immune responses. Dysbiosis during this window, whether due to cesarean section, formula feeding, or antibiotics, has been associated with an increased risk of allergic, autoimmune, and metabolic disorders [8,54,55].

Importantly, while the microbiota remains plastic during infancy and early childhood, early perturbations may lead to long-term alterations in microbial architecture and function [52,56].

**Figure 1:**
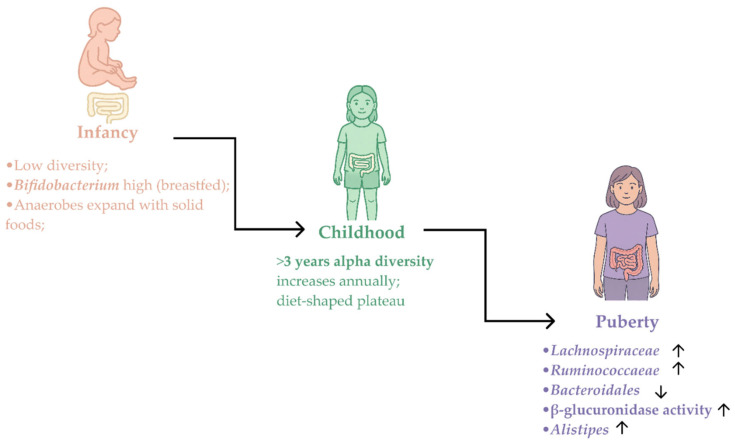
Lifespan trajectory of the gut microbiota. Infancy is characterized by low diversity with *Bifidobacterium* dominance in breastfed infants [8,55]; childhood shows high alpha diversity and a diet-shaped plateau [52]; during puberty (girls) there is enrichment of *Clostridiales*, increases in *Lachnospiraceae*/*Ruminococcaceae*, a subset-specific decline in *Bacteroidales*, and higher fecal β-glucuronidase activity, with *Alistipes* increasing [4,58,62].

From childhood to adolescence, the GM switches from a diet-shaped ‘plateau’ to a community that also reflects endocrine maturation (Figure 1). In school-age children the alpha diversity is already high and often remains stable or rises slightly as diets modify; moreover, the beta-diversity tends to widen with diverging eating patterns, adiposity and activity levels [57]. As girls enter puberty, sex steroids (especially estradiol activity), insulin/leptin mechanisms and IGF-1 reshape the intestinal milieu and a set of reproducible microbial tendencies emerges across cohorts: the community shifts towards Firmicutes-rich families (*Lachnospiraceae* and *Ruminococcaceae*). Some studies also note that the dominant taxa are *Clostridia* and *Bacteroidia* and the major element suggesting maturation of the microbiota is represented by a switch in the dominance of the *Clostridiales* and *Bacteroidales* classes of bacteria. During puberty, there is a progressive enrichment of *Clostridiales* accompanied by a reduction in *Bacteroidales*, bringing the gut microbiota closer to the adult composition. At the phylum level, *Firmicutes* increase across puberty, whereas *Bacteroidetes* decline [58,59,60,61].

The estrobolome, the set of microbial functions that deconjugate estrogens, also shows pubertal dynamics. Several pediatric cohorts report higher fecal β-glucuronidase activity during puberty, particularly when pathobionts such as *Klebsiella* and *Escherichia*/*Shigella* expand. Increased deconjugation favors intestinal reabsorption of estrogens via the enterohepatic cycle, potentially amplifying estrogen exposure once central activation has begun. This estrobolome imprint sits alongside SCFA changes and adds an endocrine-proximal mechanism by which the gut community can influence pubertal physiology [4,58].

Taken together, the physiologic maturation of the gut microbiota in girls marked by a modest shift toward *Firmicutes*, a relative decline of *Bacteroides* in a subset and *Lachnospiraceae*/*Ruminococcaceae* genera, and a measurable rise in fecal β-glucuronidase sets the background against which ICPP emerges. Case–control studies show the same axes in CPP but earlier and with greater amplitude (*Bacteroides* ↓, *Alistipes* ↑, cohort-variable *Faecalibacterium*/*Roseburia*, β-glucuronidase ↑). This amplification aligns CPP with pubertal physiology while departing from age norms, and it supports the use of stool-based, puberty-proximal features as early risk indicators [62,63,64,65,66].

## 5. Mechanistic Pathways Linking Gut Microbiota to Pubertal Regulation

The gut microbiota exerts profound systemic effects on host physiology, extending well beyond the gastrointestinal tract. In recent years, growing evidence has highlighted its potential role in modulating the HPG axis, suggesting a key function in regulating pubertal timing and progression. Several mechanistic pathways have been proposed through which the gut microbiota may influence endocrine, immune, and metabolic systems involved in pubertal development, particularly in the context of CPP [67,68,69].

Among the various mechanisms proposed to link the gut microbiota with pubertal regulation, two pathways have emerged as particularly relevant in the female pubertal trajectory: the estrobolome, through its influence on estrogen metabolism, and microbial production of SCFAs, which act as endocrine modulators of neuroimmune and hypothalamic function [70,71]. These interconnected axes represent the most biologically plausible and currently best supported mechanisms explaining microbiota-driven modulation of pubertal timing [72,73].

### 5.1. The Estrobolome and Estrogen Metabolism

The estrobolome represents the subset of the gut microbiota that possesses the capacity to metabolize estrogens primarily through the action of bacterial β-glucuronidase enzymes. These enzymes deconjugate estrogen conjugates that have been metabolized by the liver and excreted into the bile, allowing free estrogens to be reabsorbed into the circulation via enterohepatic recirculation. This microbial function plays a critical role in maintaining systemic estrogen homeostasis [58,60].

In physiological conditions, estrogens are metabolized hepatically through conjugation which facilitates their excretion into the intestinal lumen via bile. However, in the colon, β-glucuronidase-expressing bacteria, for example species within the genera *Bacteroides*, *Clostridium*, *Escherichia*, and *Lactobacillus,* can reverse this process, liberating active estrogens [52]. These unconjugated estrogens can then be reabsorbed across the intestinal epithelium, re-entering systemic circulation and contributing to the bioavailable estrogen. This mechanism of microbial-mediated estrogen recycling has been well documented in adult women, where it contributes to hormonal balance and may be dysregulated in conditions such as estrogen-sensitive cancers and metabolic syndrome [58,62]. However, its relevance to pubertal timing is now becoming increasingly apparent, particularly in the context of CPP, where estrogen exposure is prematurely elevated. In fact, studies comparing the gut microbiota of girls with ICPP to age-matched prepubertal controls have identified increased abundance of *Escherichia coli* and *Clostridium* spp., genera known to harbor high β-glucuronidase activity [52,74]. Although the directionality and causality of this relationship remain to be clarified, such findings support the hypothesis that an overactive estrobolome may contribute to premature estrogen-driven maturation.

The activity of the estrobolome is not solely dependent on microbial composition, but also on host and environmental factors. Dietary components such as fiber, phytoestrogens, and polyphenols can modulate the activity and expression of β-glucuronidase [5,75]. Moreover, early-life exposure to antibiotics, which disrupt microbial diversity and reduce β-glucuronidase-expressing taxa, has been associated with both delayed and accelerated pubertal patterns, further underscoring the complexity of the microbiota–estrogen axis [9]. Additionally, inflammatory signals in the gut microenvironment may influence the estrobolome through epigenetic regulation of bacterial gene expression or shifts in metabolic niche competition [76]. For example, inflammatory conditions such as obesity or dysbiosis-induced gut permeability can alter the functional capacity of the microbiota, leading to increased estrogen recirculation in a pro-inflammatory setting, which may further sensitize the HPG axis [22,25,77].

### 5.2. Short Chain Fatty Acids (SCFAs) and Neuroendocrine Modulation

SCFAs are small organic molecules generated mainly through the bacterial fermentation of dietary carbohydrates. The most abundant are acetate, propionate, and butyrate, while isobutyrate, valerate, and isovalerate are produced in smaller amounts. Their biosynthesis is largely attributed to colonic taxa such as *Bifidobacterium*, *Lactobacillus*, *Roseburia*, *Coprococcus*, *Faecalibacterium*, and members of the *Lachnospiraceae* family [65]. Beyond serving as an energy source for colonocytes, SCFAs exert wide-ranging effects on host physiology, including pathways relevant to pubertal development [78].

Mechanistically, SCFAs interact with the gut–brain axis by binding to receptors on intestinal endocrine cells, immune cells, and neurons, thereby influencing host gene expression. One key target is the regulation of genes encoding cAMP response element–binding proteins, which in turn modulate catecholamine biosynthesis. This includes enhancing tyrosine hydroxylase activity (the rate-limiting step in dopamine synthesis) while reducing expression of dopamine β-hydroxylase, the enzyme responsible for converting dopamine into norepinephrine. As a result, SCFAs can shape dopaminergic tone, a pathway closely linked with hypothalamic regulation of reproduction [16,17]. In addition, they modulate neurotransmitter signaling more broadly, including serotonin and dopamine, providing another layer of interaction between the gut microbiota and the central neuroendocrine circuits governing pubertal timing [78].

Among the best characterized SCFAs, butyrate stands out due to its pleiotropic effects on host physiology, ranging from energy metabolism and obesity to immune regulation, neural function and carcinogenesis. This metabolite is mainly produced by colonic bacteria such as *Faecalibacterium prausnitzii* (Clostridium cluster IV) and *Roseburia* spp. (Clostridium cluster XIV), though butyrate-producing capacity is also distributed across other clusters, including IX, XV, XVI and XVII [52].

**Figure 2:**
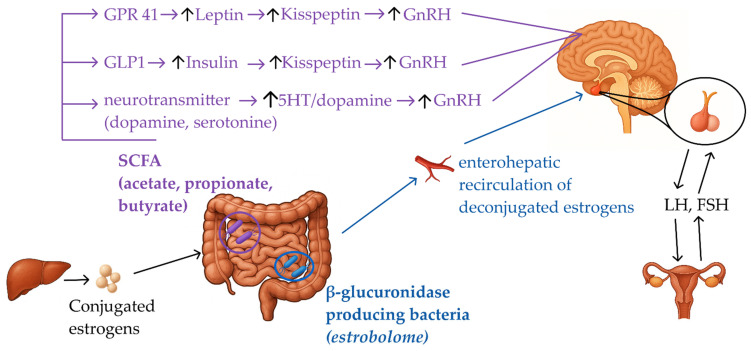
Proposed mechanisms linking gut microbiota to early pubertal onset.

Perturbations in butyrate-producing populations have been implicated in altered timing through several mechanisms. First, butyrate binds preferentially to G-protein coupled receptor 41 (GPR41), which enhances leptin expression. Leptin, in turn, regulates kisspeptin neurons and facilitates the pulsatile secretion of GnRH, a central driver of pubertal initiation [79,80]. Second mechanism, through epigenetic pathways, butyrate increases the proportion of cholinergic enteric neurons, thereby modulating acetylcholine signaling and adding another layer of gut–brain communication relevant to pubertal onset [81]. The third one, butyrate stimulates glucagon-like peptide-1 (GLP-1) release, leading to increased insulin secretion; insulin subsequently promotes GnRH gene transcription in the hypothalamus (Figure 2) [78].

While butyrate has attracted particular attention, the overall balance between the major SCFAs (acetate, propionate, and butyrate) also plays a crucial role in host physiology and may influence pubertal development [78]. These metabolites are produced in varying proportions depending on diet, microbiota composition, and metabolic status. Acetate and propionate are strongly linked to energy harvest and lipogenesis, and elevated levels have been associated with obesity and low-grade systemic inflammation, both of which are known modulators of pubertal timing [12]. In contrast, butyrate exerts predominantly anti-inflammatory and epigenetic effects that support intestinal and neuroendocrine homeostasis [82,83]. Thus, an imbalance characterized by relative depletion of butyrate-producing taxa and excess acetate/propionate production may create a pro-inflammatory and insulin-resistant milieu that facilitates premature activation of the hypothalamic–pituitary–gonadal axis. This highlights that not only the absolute levels of specific SCFAs, but also their ratios, are likely to be critical determinants linking gut microbial metabolism with the timing of puberty [12].

Although mechanistic studies suggest that butyrate may directly stimulate neuroendocrine pathways involved in pubertal initiation, clinical data in girls with CPP point to a different pattern. Case–control analyses consistently report a reduction in butyrate-producing taxa (*Faecalibacterium*, *Coprococcus*) accompanied by elevated acetate levels [64]. This imbalance results in the loss of butyrate’s protective anti-inflammatory and epigenetic functions, while excess acetate favors a pro-inflammatory and insulin-resistant state. Together, these changes create a permissive environment for premature activation of the hypothalamic–pituitary–gonadal axis, thus providing a plausible link between gut microbiota composition and altered pubertal timing in CPP. These insights underscore the potential of SCFA profiling as a non-invasive biomarker approach in assessing pubertal risk and pave the way for therapeutic interventions targeting the gut microbiome [64,78].

## 6. Gut Microbiota and Pubertal Onset-Emerging Links and Mechanistic Insights

In recent years, a growing body of literature has explored the intersection between GM and pubertal timing, particularly in the context of CPP. Although methodologies, study populations and outcomes vary, a number of shared microbial patterns and functional pathways have begun to emerge. Notably, alterations in SCFA dynamics, enrichment of bacteria with β-glucuronidase activity, and broader functional reprogramming of the GM have been reported in association with early activation of the HPG axis.

This chapter offers a narrative synthesis of evidence from both human and animal studies, aiming to bridge clinical observations with mechanistic insights. While human cohorts provide ecological relevance and highlight the complexity of real-life exposures, experimental models allow controlled dissection of microbiota-host interactions. By examining common themes, such as microbial shifts in SCFA-producing taxa, enhanced estrogen recycling and neuroendocrine modulation via microbial metabolites we aim to clarify the potential role of the GM as an upstream mediator of pubertal onset. Understanding how microbial signals converge on the HPG axis through dietary, metabolic, and immunological pathways not only sharpens our view of pubertal physiology, but also opens promising avenues for novel, microbiota-targeted strategies in the management or prevention of CPP.

### 6.1. Microbial Signatures and Mechanistic Insights in Girls with Central Precocious Puberty

Although the etiology of CPP is gradually becoming elucidated, the condition remains idiopathic in approximately 90% of cases, with no clear underlying cause identified. This has led researchers to explore emerging biological pathways that may contribute to its development beyond classical neuroendocrine models. Among these, the intestinal microbiota has attracted increasing attention, as accumulating clinical investigations suggest a consistent association between microbial dysbiosis and early activation of the HPG axis.

Emerging microbiome-metabolome profiling has revealed disinct compositional and functional changes in the GM of girls with CPP, suggesting potential microbial contributions to early endocrine activation. In one cohort, increased microbial diversity and a shift toward a more adult-like composition were observed in affected children. This configuration included the enrichment of several *Bacteroides* species (*B. vulgatus*, *B. massiliensis*, *B. cellulosilyticus*, *B. ovatus*) and other short-chain fatty acid (SCFA)-producing bacteria, such as *Parabacteroides merdae*, *Butyricicoccus*, *Ruminococcus*, and *Erysipelatoclostridium ramosum*. Notably, these microbial shifts correlated positively with elevated levels of LH and FSH, suggesting a potential role in premature activation of the HPG axis. By contrast, microbial configurations characterized by *Bacteroides dorei* and Prevotella-dominated clusters typically linked to diets rich in complex carbohydrates and seafood, were associated with lower estradiol levels and delayed bone maturation [55]. These results indicate that precocious puberty is accompanied not by a loss, but by an expansion of SCFA-producing taxa and by distinct microbiota–metabolite networks that can either accelerate or protect against early pubertal onset.

However, contrasting profiles have also been reported. In a large pediatric cohort stratified by controls, precocious puberty (PP), and obesity-complicated precocious puberty (OPP) 16S rRNA sequencing revealed a pronounced reduction in several beneficial butyrate-producing genera, including *Faecalibacterium*, *Anaerostipes*, *Bacteroides*, and *Bifidobacterium*, accompanied by significantly lower fecal SCFA concentrations in the PP and OPP groups. Importantly, the depletion of these commensals was strongly associated with higher estradiol, LH, and FSH levels, suggesting that loss of SCFA-mediated anti-inflammatory and epigenetic regulation may create a permissive environment for early HPG-axis activation [8].

A more detailed perspective on the microbial-endocrine interface in CPP comes from the study by Li et al. (2021) which explored GM composition and function across three pediatric groups: girls with central precocious puberty (CPP), overweight peers, and healthy controls [45]. In the CPP group, microbial α-diversity was notably elevated, accompanied by increased abundance of genera such as *Alistipes*, *Klebsiella*, and *Sutterella*. Interestingly, both the CPP and overweight groups showed a shared overrepresentation of *Prevotella*, suggesting a possible link between metabolic phenotype and microbial composition. Network analysis revealed denser microbial co-occurrence patterns in CPP, dominated by *Firmicutes*. Functional predictions further showed upregulation of microbial modules involved in nitric oxide, dopamine, and acetate synthesis, with nitric oxide production positively correlated with serum FSH and insulin levels. Further associations emerged between *Parabacteroides* abundance and hypothalamic LHRH (luteinizing hormone-releasing hormone), while *Akkermansia* levels correlated negatively with LH and FSH concentrations. Together, these findings underscore that CPP is marked not only by taxonomic shifts but also by functional microbial reprogramming that may intersect with host neuroendocrine pathways [45].

Emerging evidence increasingly highlights functional dimensions of gut dysbiosis in CPP. One investigation focusing on microbial payhways revealed that girls with CPP exhibited an enrichment in genes involved in cell motility, environmental adaptation, and signal transduction. Moreover, individual taxa showed distinct endocrine correlations: *Fusobacterium* abundance was positively correlated with FSH levels, while *Gemmiger* correlated with elevated LH concentrations [49]. These findings suggest that microbial communities may influence reproductive endocrinology through mechanisms beyond SCFA production, possibly involving immune modulation, metabolite signaling, or gut–brain communication.

Additional mechanistic insight comes from profiling the “estrobolome”. In girls with CPP, fecal β-glucuronidase activity was significantly elevated, promoting deconjugation and systemic reabsorption of estrogen metabolites through enterohepatic recirculation. This microbial mechanism likely amplifies systemic estrogen exposure and contributes to accelerated pubertal progression [52]. Together, these findings emphasize the need to consider microbial function, not just composition, when exploring the gut-endocrine interface in precocious puberty.

A longitudinal lens further reinforces the dynamic interplay between gut microbiota and pubertal development. Observations from a cohort of girls aged 6 to 12 revealed that, under physiological conditions, the gut microbiome undergoes progressive remodeling in parallel with hormonal maturation. As puberty unfolds, microbial communities gradually shift toward a more adult-like configuration, guided by evolving dietary habits and endocrine signals. This sincrony between microbiota and endocrine maturation appears to be a hallmark of normal pubertal timing. However, its disruption, whether by environmental, dietary, or microbial perturbations may disturb this natural alignament and create conditions conducive to premature activation of reproductive axis [11].

To reconcile the partially discordant findings across human and preclinical cohorts, the recent literature has sought to integrate key microbial signatures linked to CPP [84]. Across multiple studies, including both human and animal data, recurrent taxonomic patterns have emerged. Genera such as *Alistipes*, *Ruminococcus*, *Dialister*, *Bilophila*, and *Lachnoclostridium* tend to be enriched in CPP, whereas *Bacteroides*, *Anaerostipes*, *Megamonas*, and *Gemella* are commonly depleted. A notable trend is the increased alpha diversity observed in girls with CPP, in contrast to decreased diversity reported in animal models, underscoring the complexity of translating mechanistic findings to clinical contexts.

Beyond taxonomy, multiple studies highlight a consistent reduction in fecal SCFAs, particularly butyrate and propionate, supporting the view that compromised SCFA availability may serve as a unifying mechanism linking dysbiosis to premature activation of the HPG axis. In parallel, enrichment of bacteria harboring β-glucuronidase activity, such as *Alistipes*, *Ruminococcus*, and *Roseburia*, suggests that estrogen deconjugation and enhanced enterohepatic recirculation may amplify systemic exposure to active estrogens, providing a plausible mechanistic link to accelerated pubertal onset.

Several cohorts also report distinct microbial patterns in CPP versus normal pubertal progression, suggesting that the observed shifts may reflect independent developmental trajectories rather than a mere acceleration of typical pubertal events. Particularly telling are opposing trends such as *Bacteroides* depletion and *Ruminococcus* enrichment, potentially indicating a broader reorganization of gut microbial niches.

Taken together, three dominant themes emerge across human studies. First, altered SCFA dynamics: the loss of key butyrate-producing taxa like *Faecalibacterium* and *Anaerostipes* reduces SCFA availability and associated anti-inflammatory and epigenetic benefits [8]. Conversely, some studies show expansion of alternative SCFA producers such as *Bacteroides*, *Parabacteroides merdae*, *Butyricicoccus*, and *Ruminococcus*, hinting at functional compensation through distinct metabolic routes [55].

Second, enhanced estrogen recycling: enrichment of taxa with β-glucuronidase activity, such as *Klebsiella*, *Escherichia*, and specific *Clostridia*, supports increased deconjugation and enterohepatic recirculation of estrogens, thus potentially raising systemic estrogen levels [52].

Third, functional reprogramming: beyond compositional shifts, several studies describe enrichment in microbial pathways related to motility, environmental adaptation, and signal transduction. Taxa like *Fusobacterium* and *Gemmiger* show positive correlations with FSH and LH levels, implying direct microbe–host signaling links to the endocrine system [45].

Despite cohort-specific differences, the overarching picture is consistent: the gut microbiota in CPP acts not as a passive correlate but as a dynamic modulator of pubertal timing. Dysbiosis may manifest through loss of beneficial taxa, expansion of estrogen-activating or inflammatory microbes, or via reconfiguration of microbial functions. These ecological changes ultimately converge on common endocrine outcomes: enhanced estrogen exposure, disrupted SCFA balance, and early HPG-axis activation. Such findings position gut microbial signatures as promising non-invasive biomarkers for early puberty and suggest future opportunities for microbiota-based interventions.

To support the thematic synthesis presented above, Table 1 offers an integrative snapshot of selected human and animal studies investigating gut microbiota changes in relation to precocious puberty. Rather than providing a comprehensive survey, it highlights illustrative findings that reflect the compositional shifts and functional pathways discussed throughout the review.

### 6.2. Microbial Signals in Motion: Insights from Animal Models of Pubertal Acceleration

Animal models have been instrumental in dissecting the mechanistic links between gut microbiota, microbial metabolites, and the regulation of pubertal onset. While human studies provide associative evidence, experimental manipulations in rodents allow for causal inferences, offering critical insights into how dietary, microbial, and metabolic factors interact with the HPG axis.

Diet induced models of early puberty have been particularly revealing in exploring how microbial metabolites interact with neuroendocrine regulators. In mice exposed to a high-fat diet (HFD), a condition known to induce early pubertal onset, supplementation with SCFAs was found to counteract the pubertal acceleration typically observed under obesogenic conditions. Mechanistically, SCFAs acted through GPR41, leading to suppression of GnRH signaling [78]. These findings directly implicate SCFAs as modulators of the central reproductive axis and provide strong causal evidence that microbial metabolites can counteract diet-induced endocrine disruption.

Interestingly, subsequent investigations have revealed that SCFA activity is not universally protective. In a different experimental context, HFD feeding led to the overgrowth of bacterial taxa such as *Streptococcus* GCA 900066575, *Anaerotruncus*, and *Lachnoclostridium*, which were associated with elevated production of butyrate and proprionate. These increased SCFA levels correlated with earlier pubertal onset, suggesting that excessive SCFA accumulation, particularly within a dysbiotic microbial environment, may instead promote HPG axis activation [85]. Together, these findings underscore the context-dependent nature of SCFA signaling: while physiologic levels may help restore endocrine balance, their overproduction may paradoxically act as a pro-pubertal stimulus. This duality highlights the complexity of microbe-host interactions and cautions against viewing SCFAs as uniformly beneficial in the regulation of pubertal timing.

Maternal diet also emerged as a powerful early-life determinant of pubertal timing, acting through long-term programming of the gut microbiota and its metabolic output. In offspring of HFD-fed dams, distinct microbiota alterations were observed, including the enrichment of *Bacteroides*, *Klebsiella*, and *Helicobacter*, coupled with significant reductions in acetate, propionate, and hexanoic acid. Although butyrate also trended downward, this change was not statistically significant. These compositional and metabolic changes were accompanied by hypothalamic modifications that primed early GnRH neuron activation, illustrating how maternal nutrition can durably reshape the microbiota–metabolite axis and predispose offspring to precocious puberty [15].

Extending the line of evidence, other experiments have begun to map more direct biochemical pathways connecting gut microbes to the hypothalamic regulation of puberty. One notable study uncovered a microbiota–metabolite–KISS1 signaling pathway in which HFD exposure enriched taxa such as *Lactobacillus* and *Romboutsia*, taxa capable of producing 23-nordeoxycholic acid (norDCA) and psoralidin. These metabolites upregulated hypothalamic KISS1 expression, stimulated GnRH release, and elevated LH/FSH secretion, thereby accelerating pubertal onset. Strikingly, antibiotic-mediated disruption of the GM reversed this cascade: *Lactobacillus* and *Romboutsia* declined, KISS1 and GnRH expression decreased, and SIRT1 levels rose, ultimately alleviating precocious puberty [86]. This provides compelling evidence that specific microbial metabolites can act directly on central neuroendocrine regulators to influence reproductive timing.

Viewed as a whole, animal models provide more than experimental validation, they offer a window into the dynamic and multifaceted dialog between GM and the neuroendocrine system. These studies reveal not only how microbial communities can shape reproductive timing, but also how this influence varies depending on developmental context and metabolic state. By tracing the pathways from microbial shifts to hormonal outputs, preclinical research enriches our conceptual framework of puberty, suggesting that the gut may act less as a passive environment and more as a regulatory organ in its own right.

### 6.3. Two Roads to the Same Axis: Human and Rodent Perspectives on Puberty

Integrating evidence from both human and animal studies provides a unique lens through which the complex relationship between GM and pubertal timing can be explored. While each research approach brings its own strengths and limitations, together they contribute to a more nuanced understanding of CPP. Human cohorts capture real-world variability, where diet, antibiotic use, adiposity, and environmental factors interact to shape the microbiome. Findings such as the depletion of butyrate-producing taxa or enhanced estrobolome activity reflect this multifactorial reality [8,52,55]. In contrast, animal models rely on controlled manipulations-HFD, SCFA and antibiotic-induced dysbiosis—that isolate causal relationships but inevitably simplify the ecological complexity of the human gut [15,78,85,86].

Temporal resolution also sets these approaches apart. Most human studies are cross-sectional, offering only a snapshot of microbial composition and limiting causal inference. Longitudinal data remain scarce, through some suggest that GM maturation parallels hormonal development [11]. Rodent models, however, allow continuous monitoring throughout pubertal transition, enabling researchers to trace microbial and endocrine shifts from prepuberty through sexual maturation. [15,78,85,86].

Mechanistic insights are another domain where these paths diverge. Human data primarily highlight associations: reduced SCFA levels [8,55], expansion of β-glucuronidase–positive taxa [52], and correlations between microbial taxa and gonadotropins [49]. While informative, such findings stop short of establishing direct causality. By contrast, animal studies move beyond correlation. SCFAs supplementation has been shown to reverse diet-induced precocity [78], but in other contexts excessive SCFA production accelerated puberty [85]. This apparent paradox highlights the context-dependent role of SCFAs: physiological levels may exert protective, homeostatic functions, whereas abnormal accumulation within a dysbiotic ecosystem may trigger premature activation of the axis. Beyond SCFAs, animal work has identified additional mechanisms absent from human studies. Maternal diet reprograms the offspring microbiota and its metabolites, altering SCFA availability and pubertal timing [15]. Even more direct evidence points to specific microbial metabolites, like bile acid derivatives (norDCA) and phytoestrogen-like compounds (psoralidin) that modulate hypothalamic KISS1 expression, GnRH release, and gonadotropin secretion [86].

**Figure 3:**
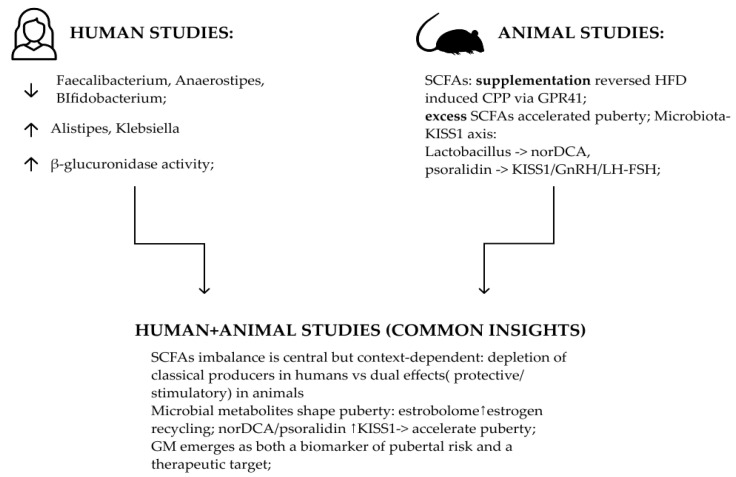
Human and animal evidence: SCFA imbalance and microbial metabolites drive early HPG-axis activation.

Despite their methodological differences, human and animal data converge on a central premise: the gut microbiota operates as both sensor and mediator of environmental signals that influence reproductive maturation (Figure 3). While human data reveal dysbiosis-associated endocrine changes [8,38,40,41], animal models demonstrate causality through interventions that restore balance or directly modulate neuroendocrine signaling [15,51,55,56], highlighting the microbiota as an upstream regulator of pubertal timing but also the need for caution when extrapolating across species. For an overview of representative taxa reported as altered in CPP, focusing on those with functional or endocrine relevance see Table 2.

Table 2 summarizes genera reported as altered in central precocious puberty (CPP) across published studies. Direction of change refers to relative abundance compared with age-matched controls. Functional relevance derived from CPP studies were reported, otherwise summarized from broader microbiome.

### 6.4. Discussion

CPP is associated with adverse mental and physical health outcomes, including higher risks of obesity, metabolic disorders, and cardiovascular disease [79]. Emerging evidence implicates the gut microbiota as a key factor in the development of CPP in girls [3,63]. In this study, we assess the relationship between GM and CPP and demonstrate that microbial composition and functional potential in CPP differ significantly from those in healthy controls. Alpha diversity appears higher in girls with CPP, echoing previous observations [79]. We also observed similarities between the GM of CPP girls and that reported in individuals with obesity [80,87].

Across included studies, beneficial bacteria such as *Bacteroides* were consistently reported to be reduced in CPP girls [79]. As principal propionate producers, *Bacteroides* play a key role in fermenting plant polysaccharides that humans cannot digest directly [88,89], and their depletion may lead to reduced availability of this antilipogenetic and antiinflamatory metabolite [90,91]. Experimental data suggest that propionate can inhibit ghrelin secretion through FFAR2/FFAR3 signaling [92]. Consequently, reduced propionate may allow ghrelin levels to rise. Higher circulating ghrelin, in turn, has the potential to stimulate GnRH neuronal activity, increase LH pulsatility, and promote estradiol production [76,93]. Taken together, this sequence provides a plausible pathway by which reduced *Bacteroides* and lower propionate availability might indirectly contribute to the earlier onset of pubertal development [94]. Beyond their metabolic contributions, *Bacteroides* also appear to mark critical stages of gut microbial maturation. Previous studies have shown that *Bacteroides* becomes a dominant genus during the transition from infancy to adulthood, shaping a more diverse and interconnected microbial ecosystem [55,95]. In this context, the consistent depletion of *Bacteroides* in CPP may reflect a disrupted maturation trajectory of the gut microbiome, potentially decoupling microbial development from neuroendocrine progression.

In addition to its role in ghrelin regulation, propionate has also been shown to enhance satiety by stimulating the release of gut hormones such as peptide YY (PYY) and glucagon-like peptide-1 (GLP-1), independently of FFAR3 signaling [92]. These hormones contribute to appetite regulation, glucose homeostasis, and gut–brain communication, pathways increasingly recognized as relevant to pubertal onset. Therefore, a sustained reduction in propionate availability could disrupt not only metabolic feedback loops, but also neuroendocrine circuits essential for pubertal timing.

Based on the reduction in beneficial taxa like *Bacteroides*, we examined the Firmicutes to Bacteroidetes (F/B) ratio and found it elevated in CPP compared to controls [79]. An elevated F/B ratio typically reflects reduced saccharolytic capacity and lower SCFA output, with downstream effects on enteroendocrine signaling (e.g., PYY, GLP-1), vagal input, intestinal barrier integrity, and low-grade inflammation [80,96]. Collectively, these microbial shifts offer a biologically plausible route to earlier activation of hypothalamic kisspeptin/GnRH networks and increased LH pulsatile activity, accelerating pubertal timing in idiopathic CPP.

While the F/B ratio can be influenced by diet, BMI, and stool form, in our dataset it mirrors the genus-level decrease in *Bacteroides* and aligns with functional readouts such as SCFA and estrobolome alterations [79]. Thus, F/B ratio may be better considered an ecosystem-level marker rather than a standalone diagnostic. Notably, similar F/B alterations have been reported in pediatric obesity [80,96]. However, in CPP, this microbial signature appears tied to pubertal activation itself, not merely to adiposity.

#### 6.4.1. Key Microbial Genera and Functional Outputs Implicated in Pubertal Acceleration

One of the most reproducible findings across human studies is the depletion of *Bacteroides*, a major propionate producer [52]. Reduced propionate availability has been linked to altered ghrelin signaling [4], a pathway already implicated in early HPG-axis activation.

In contrast, the behavior of butyrate-producing taxa such as *Faecalibacterium*, *Roseburia*, and *Ruminococcus* is less uniform. Some cohorts describe their depletion, others an enrichment [52,97]. These discrepancies most likely reflect differences in host diet, BMI, geography, and sequencing methodology rather than true biological contradictions. Importantly, butyrate producers are embedded in a network of cross-feeding interactions. For example, taxa like *Alistipes* contribute acetate and succinate, which can be metabolized by *Faecalibacterium* and *Roseburia* into butyrate [98]. Thus, enrichment of butyrate producers in some studies may represent a compensatory adjustment within the microbial ecosystem, not a conflict with the propionate-deficit model.

One genus that warrants particular attention in the context of CPP is *Alistipes*. Although not a butyrate producer, *Alistipes* has been consistently found to be enriched in Asian cohorts of girls with CPP [99] where it has been linked to mucosal inflammation, abdominal pain, and the production of metabolites such as acetate and succinate [100]. These intermediates can fuel butyrate-producing taxa, but *Alistipes* also generates neuroactive compounds that influence serotonin and dopamine signaling at the mucosal level, providing a direct mechanism for gut–brain communication and potentially contributing to early HPG axis activation [101]. However, findings from European populations suggest a different role. Some studies have associated *Alistipes* with protective effects, including the modulation of host metabolism and attenuation of high-fat-diet-induced inflammation [49,100]. Experimental evidence indicates that *Alistipes* or its metabolites may support intestinal barrier function, regulate apetite, and reduce levels of inflammatory mediators such as nitric oxide, which is known to stimulate sex hormone production [102]. These contrasting observations point to a context-dependent role of *Alistipes*, shaped by host metabolic status, ethnic background and dietary patterns. Rather than representing a universally harmful or protective taxon, *Alistipes* may act along a spectrum of effects, influenced by the ecological and physiological landscape of the host. Such complexity underscores the need for cross-cultural studies to fully elucidate the microbial mechanisms underlying CPP.

Adding to this complex microbial landscape, *Prevotella* has also emerged as a genus of interest in the context of pubertal regulation. Although more frequently studied in relation to diet and metabolic health, certain *Prevotella* species, such as *Prevotella copri*, have recently been associated with reduced estradiol levels and delayed pubertal progression [55]. These bacteria possess the enzymatic capacity to modulate steroid metabolism, potentially enhancing the catabolism of estrogens. Interestingly, increased *Prevotella* abundance has been linked to lower bone age advancement and a favorable hormonal profile in girls with early pubertal signs, suggesting a protective influence against the premature activation of the HPG axis [103]. While evidence remains preliminary, these findings underscore the potential of specific microbial configurations to counterbalance endocrine disruptions in CPP.

Beyond endocrine pathways, oxidative stress has emerged as an additional factor that may link gut microbial shifts to pubertal timing. Research suggests that puberty is associated with increased production of reactive oxygen species (ROS), which drive lipid peroxidation and the accumulation of oxidative metabolites such as 13-KODE and 9(R)-HODE [55]. These markers have been found elevated in children with CPP, reinforcing the notion that redox imbalance may accompany or contribute to early pubertal onset. Of particular interest is nitric oxide (NO), a downstream product of inflammatory and oxidative processes, which functions as a neuromodulator and has been shown to enhance GnRH release [104]. In this context, microbiota-driven changes that promote oxidative stress, either through altered SCFA balance, immune activation, or metabolite signaling, could potentiate HPG-axis activation via NO-sensitive pathways.

From a clinical standpoint, these insights highlight the potential of gut microbiota profiling as a complementary tool in assessing pubertal risk. The consistent depletion of *Bacteroides* and the resulting propionate deficit, alongside context-dependent shifts in taxa such as *Alistipes* and *Prevotella*, suggest that specific microbial signatures may serve as early indicators—or even modulators—of neuroendocrine activation. Incorporating microbial and metabolite assessments into routine pediatric evaluations could ultimately aid in the early identification of children at risk for CPP, paving the way for microbiota-targeted preventive or therapeutic strategies.

These emerging associations, however, should be interpreted with caution. Key modulators of SCFA pathways, such as dietary fiber intake, BMI, and antibiotic exposure, were not consistently controlled across the available studies, potentially confounding observed microbiota-puberty relationships [63]. Moreover, the predominance of cross-sectional designs limits causal inference, underscoring the need for longitudinal research to validate these microbial signatures and clarify their mechanistic contributions to pubertal timing [105].

#### 6.4.2. Obesity-like Microbiome Features in CPP: Overlap and Boundaries

Childhood obesity and CPP have both shown a rising prevalence in recent years, and they frequently co-occur in clinical settings. While nutritional habits appear to be a shared upstream factor, recent evidence suggests that these two conditions may intersect mechanistically but are not pathophysiologically equivalent. Observational data confirm that unhealthy dietary patterns, particularly HFD, are significantly associated with earlier pubertal onset [106,107]. However, mounting experimental data indicate that this association is not mediated solely through increased adiposity.

A key differentiator is the role of the GM. CPP-asociated dysbiosis shares certain compositional features with obesity, such as enrichment of *Gemmiger*, *Ruminococcus*, *Oscillibacter*, and members of *Clostridium* cluster XIVb-but divereges in function [49]. At the species level, signals such as *Ruminococcus gnavus* and *Ruminococcus bromii*, previously associated with increased energy harvest and weight gain in obese individuals, have also been observed in CPP cohorts [108]. This overlap likely reflects shared metabolic pressures, including diet-related shifts and low-grade inflammation, and may partly explain the increased risk of obesity later in life among girls with early menarche [80].

Yet CPP cannot be reduced to an “obesity microbiome.” First, endocrine coupling is stronger: microbial shifts in CPP correlate with circulating LH, estradiol, and kisspeptin/GnRH activity, suggesting direct links to pubertal activation beyond adiposity [105]. Second, the SCFA profile diverges. For example, CPP is marked by depletion of *Bacteroides* and reduced propionate levels, suggesting a shift in SCFA signaling that may impact enteroendocrine pathways involved in HPG axis regulation. Similarly, while *Faecalibacterium* and *Roseburia* are butyrate producers enriched in both states, their presence in CPP often results from altered microbial cross-feeding patterns rather than classic energy-harvesting dominance. Finally, CPP-specific estrobolome activation (increased fecal β-glucuronidase) enhances estrogen recirculation—a hormonal pathway less strongly implicated in obesity [109].

More importantly, recent animal studies show that HFD can promote early puberty via microbiota-independent mechanisms. Fatty acids in HFDs have been shown to activate hypothalamic microglial cells, immune cells that interact directly with GnRH neurons via prostaglandin-mediated signaling [110,111]. This microglial activation triggers increased GnRH pulsatility, independent of body fat levels [112,113]. In fact, genetically obese mice without HFD exposure do not show microglial activation, highlighting that dietary fat, not obesity per se, drives this neuroinflammatory mechanism [85]. Additionally, HFD may induce puberty through phoenixin-mediated pathways and the overexpression of p53 via the Lin28/let-7 axis—both newly proposed links between diet and central neuroendocrine activation [114,115].

Altogether, these findings define CPP as a condition that shares part of the microbial scaffold of obesity but carries distinct endocrine signatures. Recognizing this boundary is critical: it underscores why girls with CPP cannot be evaluated solely through the lens of adiposity, and why microbiota analysis may provide independent biomarkers for pubertal timing [96].

## 7. Conflicting Evidence and Remaining Gaps

The current body of evidence linking gut microbiota to CPP remains fragmented and, at times, contradictory. Some cohorts consistently report a depletion of butyrate-producing taxa such as *Faecalibacterium* and *Anaerostipes* [8], while others describe an enrichment of alternative SCFA-producing species, including multiple *Bacteroides* species, *Parabacteroides*, and *Ruminococcus* [42]. This inconsistency extends to functional findings as well: although increased β-glucuronidase activity is a recurring observation, its causal role in estrogen reactivation and pubertal acceleration remains debated [41]. Such discrepancies highlight the complexity of microbiota–host interactions and emphasize the need for cautious interpretation. Adding to this complexity, recent findings indicate that PPP may represent an intermediate stage between CPP and healthy children. Overlapping alterations in gut microbiota composition have been observed in both PPP and CPP cohorts suggesting these endocrine phenotypes may reflect points along a continuum rather than distinct entities [52]. This raises important questions regarding the pathophysiological overlap and potential sequential progression between peripheral and central forms of precocious puberty, which remain insufficiently understood.

Despite growing interest, significant limitations still constrain this field. Most available human studies are cross-sectional and observational, restricting causal inference; even consistent findings such as *Bacteroides* depletion and propionate shortfall should be regarded as associations rather than proven mechanisms [40]. Human cohorts are heterogeneous in dietary background, ethnicity, and sequencing methodology (16S rRNA vs. shotgun metagenomics), which complicates direct comparison and likely contributes to conflicting results. The predominance of Asian cohorts further limits generalizability, since gut microbiota composition differs markedly across populations, with Asian children often exhibiting higher *Bifidobacterium* abundance than their Western peers [99]. Cohort sizes are also relatively small and single-center, raising concerns about statistical power and external validity [99].

Mechanistic insights, although compelling, rely heavily on animal models. While such experiments establish causality, they cannot replicate the timing and complexity of human pubertal physiology, making direct extrapolation from rodents to children problematic [15,51,55]. Additional challenges arise from uncontrolled confounders—diet, BMI, antibiotic exposure, and socioeconomic context—that blur the distinction between microbiota-specific effects and broader environmental influences [60]. Furthermore, functional readouts such as fecal SCFA levels or estrobolome activity were inconsistently assessed, despite being central to mechanistic interpretation. Finally, the absence of large, multicenter, longitudinal studies with standardized protocols integrating microbiota, metabolomics, and endocrine profiling continues to delay translation into clinical practice and hinders the development of robust biomarkers for CPP risk prediction [52].

Addressing these inconsistencies through well-designed, longitudinal, and multi-ethnic studies will be essential to determine whether gut microbiota can truly serve as a reliable biomarker for CPP risk prediction. Beyond diagnostics, confirming a causal role would also open the door to microbiota-targeted interventions such as dietary modulation, prebiotics or probiotics offering novel strategies to delay or prevent premature pubertal onset.

## 8. Future Perspectives

Advancing our understanding of the gut microbiota’s role in pubertal timing may open new therapeutic frontiers in pediatric endocrinology. While most current studies have focused on observational associations, emerging insights into microbial metabolites and host metabolic signaling suggest that the microbiota could evolve from a passive biomarker into an active therapeutic target. Strategically modifying GM composition may ultimately complement existing therapies that suppress hypothalamic activation.

One promising direction is the development of microbiota-targeted interventions, including dietary and pharmacological strategies designed to restore microbial homeostasis. Among these, dietary polyphenols, notably epigallocatechin gallate (EGCG), a green tea catechin, are of growing interest. According to Rodríguez-Daza and colleagues, certain polyphenols display a “duplibiotic” effect—a dual ability to suppress pathobionts while enriching beneficial microbes such as *Akkermansia muciniphila*. In animal models, EGCG supplementation has been shown to enhance gut barrier integrity, reduce inflammatory signaling, and increase *Akkermansia* abundance, features highly relevant to pubertal timing and endocrine balance [116,117]. Other microbiota-directed strategies, such as probiotic supplementation and fecal microbiota transplantation (FMT), also warrant consideration. While these remain experimental in pediatric populations, their potential to recalibrate host–microbiome interactions and modulate gut–brain and gut–endocrine axes may provide future adjuncts to standard endocrine therapies [78,118].

Translating these findings into clinical impact will require mechanistically driven, longitudinal studies that integrate microbiome, metabolomic, and endocrine profiling in ICPP cohorts. Moving beyond descriptive correlations, such work could redefine how we approach the prevention and treatment of early pubertal onset, positioning the gut microbiota not only as a modulator of timing, but as a modifiable therapeutic frontier.

## 9. Conclusions

This review underscores the emerging role of the gut microbiota in central precocious puberty, highlighting three recurring mechanisms: a consistent loss of propionate-producing taxa such as *Bacteroides*, enrichment of β-glucuronidase–positive microbes that enhance estrogen recirculation, and context-dependent shifts in butyrate producers. While these features overlap partially with obesity-associated dysbiosis, the CPP microbiome is distinguished by stronger endocrine coupling, reflected in links to LH/estradiol, kisspeptin/GnRH activity, and estrobolome function. From a clinical perspective, the gut microbiota should not yet be viewed as a stand-alone diagnostic marker, but stool-based microbial signatures, when combined with endocrine and clinical parameters, may provide a non-invasive tool for early risk stratification.

## Figures and Tables

**Table 1:** Representative human and animal studies exploring gut microbiota alterations in relation to precocious puberty.

Authors	Study Type	Age	Model	Method	Sample Size	Changes in GM
1. Li Wang et al. (2024) [8]	Human	6–9 years	Girls with PP vs. controls	16S rRNA gene sequencing (fecal)	CPP (*n* = 60) vs. age matched controls	↑ *Alistipes*, *Klebsiella*; ↓ *Anaerostipes*, *Bifidobacterium*, *Bacteroides* in CPP; *Anaerostipes* abundance was inversely correlated with estradiol levels
2. Wang et al. (2024) [55]	Human	5–8 years	Girls with CPP, PPP vs. normal controls	16S rRNA gene sequencing (fecal)	CPP (*n* = 21), PPP (*n* = 45) vs. age-matched controls	α-diversity ↑ (CPP and PPP vs. normal); ↑ SCFA-producing taxa (*Parabacteroides merdae*, *Butyricicoccus*, *Ruminococcus*, *Erysipelatoclostridium ramosum*); ↑ *Bacteroides* spp.; associated with early HPG axis activation
3. Li et al. (2021) [45]	Human	<8 years	CPP vs. healthy girls	16S rRNA sequencing (fecal) + functional profiling	CPP (*n* = 27) vs. age matched controls	↑ α-diversity in CPP ↑ *Klebsiella*, *Alistipes*; Network analysis showed enhanced co-occurrence in CPP (Firmicutes dominance). *Parabacteroides* positively correlated with luteinizing hormone-releasing hormone, while serotonin-producer *Akkermansia* exhibited negative relationships with FSH and LH
4. Huang et al. (2022) [52]	Human	6–10 years	CPP, PPP vs. controls	16S rRNA sequencing + fecal estrobolome assay	CPP (*n* = 27), PPP (n = 18) vs. age matched controls	↑ β-glucuronidase activity; enhanced estrogen reabsorption via estrobolome ↑ *Roseburia*, *Ruminococcus*, *Alistipes*, *Fusicatenibacter*, *Gemmiger* in CPP group
5. Dong et al. (2020) [49]	Human	<8 years	ICPP vs. healthy controls	16S rRNA sequencing (fecal) + predicted functional profiling	CPP (*n* = 25) vs. age matched controls	The GM of the CPP group was enriched for the microbial functions of cell motility, signal transductions and environmental adaptation; positive correlations were also detected between *Fusobacterium* and FSH and *Gemmiger* and LH
6. Korpella et al. (2021) [11]	Human	6–12 years	Longitudinal study, girls through puberty	16S rRNA sequencing (longitudinal profiling)	116 stool samples (girls followed over time)	Gradual microbiota shift correlates with hormonal changes across puberty
7. Wang et al. (2020) [78]	Animal	NR	Mice with HFD + SCFA treatment	16S rRNA sequencing; microbiota transplantation and SCFA intervention	HFD-exposed offspring vs. controls	The abundances of *Ruminococcaceae*, *Stretococcacea*, *Bacillacea* were higher in the maternal HFD group than in the control group;
8. Bo et al. (2022) [85]	Animal	NR	Female mice, SCFA supplementation	16S rRNA sequencing; SCFA level analysis	HFD group vs. controls	The abundances of *Streptococcus* GCA 900066575, *Anaerotruncus*, *Lachnostidrium* were higher (at the genus level) in the HFD group than in the control group; Elevated bacterial secretion of butyrate and propionate has been correlated with earlier pubertal onset
9. Wang et al. (2022) [15]	Animal	NR	Female mice, Maternal high fat diet	16S rRNA sequencing;	HFD group vs. controls	The abundances of *Bacteroides*, *Klebsiella*, *Helicobacter* were significantly higher (at the genus level) in the HFD group than in the control group; In the HFD cohort, acetic, propionic, and hexanoic acids were significantly decreased versus controls, whereas butyric acid exhibited a non-significant decline
10. Wu et al. (2025) [86]	Animal	NR	Female rats, HFD, HFD + antibiotics	16S rRNA sequencing; targeted metabolomics (bile acids and metabolites)	HFD group ± antibiotic vs. controls	Gut microbiota -> metabolites (norDCA, psoralidin) -> ↑ Kiss1 -> ↑ GnRH -> ↑ LH/FSH -> Pubertal activation

CPP—central precocious puberty; ICPP—idiopathic CPP; PPP—peripheral precocious puberty; SCFA—short-chain fatty acid; HPG—hypothalamic–pituitary–gonadal; HFD—high-fat diet; GnRH—gonadotropin-releasing hormone; LH—luteinizing hormone; FSH—follicle-stimulating hormone.

**Table 2:** Gut microbiota taxa reported as altered in central precocious puberty (CPP) across representative studies, with a focus on genera with endocrine relevance.

Taxon	Direction of Change in CPP	Functional Relevance	Supporting Evidence
*Alistipes*	*↑*	Produces acetate/succinate; Linked to inflammation Enriched in multiple cohorts	Li Wang et al. 2024 [8] Li et al. 2021 [45]
*Klebsiella*	*↑*	Β-glucuronidase activity -> estrogen reactivation	Huang et al. 2022 [52]
*Anaerostipes*	*↓*	Butyrate producer; Negatively correlated with estradiol;	Li Wang et al. 2024 [8]
*Faecalibacterium*	*↓*	Butyrate producer; Lower abundance linked to reduced SCFA	Wang et al. 2024 [55]
*Bacteroides*	*↓*	Major propionate producer	Huang et al. 2022 [52]
*Fusobacterium*	*↑*	Positively correlated with FSH	Dong et al. 2020 [49]
*Gemmiger*	*↑*	Positively correlated with LH	Dong et al. 2020 [49]
*Escherichia*	*↑*	Estrobolome activity	Huang et al. 2022 [52]

## Data Availability

No new data were created or analyzed in this study. Data sharing is not applicable to this article.

## Abbreviations

The following abbreviations are used in this manuscript:

cAMP	Cyclic adenosine monophosphate
CNS	Central nervous system
CPP	Central precocious puberty
EDCs	Endocrine-disrupting chemicals
EGCG	Epigallocatechin Gallate
FFAR2	Free fatty acid receptor 2
FFAR3	Free fatty acid receptor 3
FMT	Fecal microbiota transplatation
FSH	Follicle stimulating hormone
F/B	Firmicutes to Bacteroidetes
GLP-1	Glucagon-like peptide-1
GM	Gut microbiota
GnRH	Gonadotropin-releasing hormone
GPR41	G-protein coupled receptor 41
HFD	High-fat diet
HPG	Hypothalamic-pituitary-gonadal
ICPP	Idiopathic central precocious puberty
LH	Luteinizing hormone
LHRH	Luteinizing hormone-releasing hormone
NO	Nitric oxide
norDCA	23-nordeoxycholic acid
OPP	Obesity-complicated precocious puberty
PP	Precocious puberty
PPP	Peripheral precocious puberty
PYY	Peptide YY
ROS	Reactive oxygen species
SCFAs	Short-chain fatty acids

## References

[1] Mancini A., Magnotto J.C., Abreu A.P. (2022). Genetics of Pubertal Timing. Best Pract. Res. Clin. Endocrinol. Metab..

[2] Abreu A.P., Kaiser U.B. (2016). Pubertal Development and Regulation. Lancet Diabetes Endocrinol..

[3] Yue M., Zhang L. (2024). Exploring the Mechanistic Interplay between Gut Microbiota and Precocious Puberty: A Narrative Review. Microorganisms.

[4] Hu S., Ding Q., Zhang W., Kang M., Ma J., Zhao L. (2023). Gut Microbial Beta-Glucuronidase: A Vital Regulator in Female Estrogen Metabolism. Gut Microbes.

[5] Baker J.M., Al-Nakkash L., Herbst-Kralovetz M.M. (2017). Estrogen–Gut Microbiome Axis: Physiological and Clinical Implications. Maturitas.

[6] Qian Y., Fang X., Chen Y., Ding M., Gong M. (2024). Gut Flora Influences the Hypothalamic-Gonadal Axis to Regulate the Pathogenesis of Obesity-Associated Precocious Puberty. Sci. Rep..

[7] Kim S.J., Kim J.H., Hong Y.H., Chung I.H., Lee E.B., Kang E., Kim J., Yang A., Rhie Y.-J., Yoo E.-G. (2023). 2022 Clinical Practice Guidelines for Central Precocious Puberty of Korean Children and Adolescents. Ann. Pediatr. Endocrinol. Metab..

[8] Wang L., Yi Q., Xu H., Liu H., Tan B., Deng H., Chen Y., Wang R., Tang F., Cheng X. (2024). Alterations in the Gut Microbiota Community Are Associated with Childhood Obesity and Precocious Puberty. BMC Microbiol..

[9] Hu Y., Li J., Yuan T., Yu T., Chen Y., Kong H., Lin C., Shen Z., Tian Y., Tong S. (2022). Exposure to Antibiotics and Precocious Puberty in Children: A School-Based Cross-Sectional Study in China. Environ. Res..

[10] Tzounakou A.-M., Stathori G., Paltoglou G., Valsamakis G., Mastorakos G., Vlahos N.F., Charmandari E. (2024). Childhood Obesity, Hypothalamic Inflammation, and the Onset of Puberty: A Narrative Review. Nutrients.

[11] Korpela K., Kallio S., Salonen A., Hero M., Kukkonen A.K., Miettinen P.J., Savilahti E., Kohva E., Kariola L., Suutela M. (2021). Gut Microbiota Develop towards an Adult Profile in a Sex-Specific Manner during Puberty. Sci. Rep..

[12] You X., Yang W., Li X., Li X., Huang Y., Huang C. (2025). Dietary Modulation of Pubertal Timing: Gut Microbiota-Derived SCFAs and Neurotransmitters Orchestrate Hypothalamic Maturation via the Gut–Brain Axis. J. Endocrinol. Investig..

[13] Pedro M., Adriana D.S.L., Karolina S. (2024). Physiology of GnRH and Gonadotrophin Secretion. Endotext [Internet].

[14] Spaziani M., Tarantino C., Tahani N., Gianfrilli D., Sbardella E., Lenzi A., Radicioni A.F. (2021). Hypothalamo-Pituitary Axis and Puberty. Mol. Cell. Endocrinol..

[15] Wang L., Xu H., Tan B., Yi Q., Liu H., Deng H., Chen Y., Wang R., Tian J., Zhu J. (2022). Gut Microbiota and Its Derived SCFAs Regulate the HPGA to Reverse Obesity-Induced Precocious Puberty in Female Rats. Front. Endocrinol..

[16] Anderson G.M., Hill J.W., Kaiser U.B., Navarro V.M., Ong K.K., Perry J.R.B., Prevot V., Tena-Sempere M., Elias C.F. (2024). Metabolic Control of Puberty: 60 Years in the Footsteps of Kennedy and Mitra’s Seminal Work. Nat. Rev. Endocrinol..

[17] Navarro V.M. (2020). Metabolic Regulation of Kisspeptin—The Link between Energy Balance and Reproduction. Nat. Rev. Endocrinol..

[18] Koysombat K., Tsoutsouki J., Patel A.H., Comninos A.N., Dhillo W.S., Abbara A. (2025). Kisspeptin and Neurokinin B: Roles in Reproductive Health. Physiol. Rev..

[19] Iwasa T., Minato S., Imaizumi J., Yoshida A., Kawakita T., Yoshida K., Yamamoto Y. (2022). Effects of Low Energy Availability on Female Reproductive Function. Reprod. Med. Biol..

[20] Farooqi I.S., Jebb S.A., Langmack G., Lawrence E., Cheetham C.H., Prentice A.M., Hughes I.A., McCamish M.A., O’Rahilly S. (1999). Effects of Recombinant Leptin Therapy in a Child with Congenital Leptin Deficiency. N. Engl. J. Med..

[21] Farooqi I.S., Wangensteen T., Collins S., Kimber W., Matarese G., Keogh J.M., Lank E., Bottomley B., Lopez-Fernandez J., Ferraz-Amaro I. (2007). Clinical and Molecular Genetic Spectrum of Congenital Deficiency of the Leptin Receptor. N. Engl. J. Med..

[22] Shi L., Jiang Z., Zhang L. (2022). Childhood Obesity and Central Precocious Puberty. Front. Endocrinol..

[23] Barabás K., Szabó-Meleg E., Ábrahám I.M. (2020). Effect of Inflammation on Female Gonadotropin-Releasing Hormone (GnRH) Neurons: Mechanisms and Consequences. Int. J. Mol. Sci..

[24] Watanobe H., Hayakawa Y. (2003). Hypothalamic Interleukin-1β and Tumor Necrosis Factor-α, But Not Interleukin-6, Mediate the Endotoxin-Induced Suppression of the Reproductive Axis in Rats. Endocrinology.

[25] Lainez N.M., Coss D. (2019). Obesity, Neuroinflammation, and Reproductive Function. Endocrinology.

[26] Soliman A.T., Alaaraj N., De Sanctis V., Hamed N., Alyafei F., Ahmed S. (2023). Long-Term Health Consequences of Central Precocious/Early Puberty (CPP) and Treatment with Gn-RH Analogue: A Short Update: Long Term Consequences of Precocious Puberty. Acta Biomed. Atenei Parm..

[27] Brito V.N., Spinola-Castro A.M., Kochi C., Kopacek C., Silva P.C.A.D., Guerra-Júnior G. (2016). Central Precocious Puberty: Revisiting the Diagnosis and Therapeutic Management. Arch. Endocrinol. Metab..

[28] Cantas-Orsdemir S., Eugster E.A. (2019). Update on Central Precocious Puberty: From Etiologies to Outcomes. Expert Rev. Endocrinol. Metab..

[29] Fanaki M., Michala L., Nazari E., Daskalakis G. (2024). Central Precocious Puberty During the COVID-19 Pandemic Period: A Systematic Review of Literature. Cureus.

[30] Zhang J., Xu J., Tang X., Wu R. (2024). Comparison of Central Precocious Puberty Frequency before and during COVID-19: A Systematic Review and Meta-Analysis. BMC Endocr. Disord..

[31] Root A.W. (2000). Precocious Puberty. Pediatr. Rev..

[32] Gangat M., Radovick S. (2021). Precocious Puberty. Minerva Pediatr..

[33] Predieri B., Iughetti L., Bernasconi S., Street M.E. (2022). Endocrine Disrupting Chemicals’ Effects in Children: What We Know and What We Need to Learn?. Int. J. Mol. Sci..

[34] Zevin E.L., Eugster E.A. (2023). Central Precocious Puberty: A Review of Diagnosis, Treatment, and Outcomes. Lancet Child Adolesc. Health.

[35] Kim S.H., Huh K., Won S., Lee K.-W., Park M.-J. (2015). A Significant Increase in the Incidence of Central Precocious Puberty among Korean Girls from 2004 to 2010. PLoS ONE.

[36] Talarico V., Rodio M.B., Viscomi A., Galea E., Galati M.C., Raiola G. (2021). The Role of Pelvic Ultrasound for the Diagnosis and Management of Central Precocious Puberty: An Update. Acta Biomed. Atenei Parm..

[37] Eugster E.A. (2019). Treatment of Central Precocious Puberty. J. Endocr. Soc..

[38] Corripio R., Soriano-Guillén L., Herrero F.-J., Castro-Feijoó L., Escribano A., Sol-Ventura P., Espino R., Vela A., Labarta J.-I. (2024). The Spanish PUBERE Group; et al. Adult Height in Girls with Idiopathic Central Precocious Puberty Treated with Triptorelin. Front. Endocrinol..

[39] Sisk-Hackworth L., Kelley S.T., Thackray V.G. (2023). Sex, Puberty, and the Gut Microbiome. Reprod. Camb. Engl..

[40] Latronico A.C., Brito V.N., Carel J.-C. (2016). Causes, Diagnosis, and Treatment of Central Precocious Puberty. Lancet Diabetes Endocrinol..

[41] Pantazi A.C., Balasa A.L., Mihai C.M., Chisnoiu T., Lupu V.V., Kassim M.A.K., Mihai L., Frecus C.E., Chirila S.I., Lupu A. (2023). Development of Gut Microbiota in the First 1000 Days after Birth and Potential Interventions. Nutrients.

[42] Davis E.C., Monaco C.L., Insel R., Järvinen K.M. (2024). Gut Microbiome in the First 1000 Days and Risk for Childhood Food Allergy. Ann. Allergy. Asthma. Immunol..

[43] Yuan X., Chen R., Zhang Y., Lin X., Yang X. (2020). Gut Microbiota: Effect of Pubertal Status. BMC Microbiol..

[44] Del Castillo-Izquierdo Á., Mayneris-Perxachs J., Fernández-Real J.M. (2022). Bidirectional Relationships between the Gut Microbiome and Sexual Traits. Am. J. Physiol.-Cell Physiol..

[45] Li Y., Shen L., Huang C., Li X., Chen J., Li S.C., Shen B. (2021). Altered Nitric Oxide Induced by Gut Microbiota Reveals the Connection between Central Precocious Puberty and Obesity. Clin. Transl. Med..

[46] Mazgaj M. (2013). Diagnostyka Ultrasonograficzna Narządów Jamy Brzusznej w Przypadku Przedwczesnego Dojrzewania Płciowego u Dziewcząt. J. Ultrason..

[47] Calcaterra V., Klersy C., Vinci F., Regalbuto C., Dobbiani G., Montalbano C., Pelizzo G., Albertini R., Larizza D. (2020). Rapid Progressive Central Precocious Puberty: Diagnostic and Predictive Value of Basal Sex Hormone Levels and Pelvic Ultrasound. J. Pediatr. Endocrinol. Metab..

[48] Santos-Marcos J.A., Mora-Ortiz M., Tena-Sempere M., Lopez-Miranda J., Camargo A. (2023). Interaction between Gut Microbiota and Sex Hormones and Their Relation to Sexual Dimorphism in Metabolic Diseases. Biol. Sex Differ..

[49] Dong G., Zhang J., Yang Z., Feng X., Li J., Li D., Huang M., Li Y., Qiu M., Lu X. (2020). The Association of Gut Microbiota With Idiopathic Central Precocious Puberty in Girls. Front. Endocrinol..

[50] Bangalore Krishna K., Fuqua J.S., Rogol A.D., Klein K.O., Popovic J., Houk C.P., Charmandari E., Lee P.A. (2019). Use of Gonadotropin-Releasing Hormone Analogs in Children: Update by an International Consortium. Horm. Res. Paediatr..

[51] Yeh S.-N., Ting W.-H., Huang C.-Y., Huang S.-K., Lee Y.-C., Chua W.-K., Lin C.-H., Cheng B.-W., Lee Y.-J. (2021). Diagnostic Evaluation of Central Precocious Puberty in Girls. Pediatr. Neonatol..

[52] Huang C., Liu H., Yang W., Li Y., Wu B., Chen J., Yang Z., Liao C., Liu L., Zhang X. (2022). Distinct Gut Microbiota Structure and Function of Children with Idiopathic Central and Peripheral Precocious Puberty. Int. J. Endocrinol..

[53] Yatsunenko T., Rey F.E., Manary M.J., Trehan I., Dominguez-Bello M.G., Contreras M., Magris M., Hidalgo G., Baldassano R.N., Anokhin A.P. (2012). Human Gut Microbiome Viewed across Age and Geography. Nature.

[54] Xu C., Zhu H., Qiu P. (2019). Aging Progression of Human Gut Microbiota. BMC Microbiol..

[55] Wang Y., Jin C., Li H., Liang X., Zhao C., Wu N., Yue M., Zhao L., Yu H., Wang Q. (2024). Gut Microbiota-Metabolite Interactions Meditate the Effect of Dietary Patterns on Precocious Puberty. iScience.

[56] Strati F., Di Paola M., Stefanini I., Albanese D., Rizzetto L., Lionetti P., Calabrò A., Jousson O., Donati C., Cavalieri D. (2016). Age and Gender Affect the Composition of Fungal Population of the Human Gastrointestinal Tract. Front. Microbiol..

[57] Ringel-Kulka T., Cheng J., Ringel Y., Salojärvi J., Carroll I., Palva A., De Vos W.M., Satokari R. (2013). Intestinal Microbiota in Healthy U.S. Young Children and Adults—A High Throughput Microarray Analysis. PLoS ONE.

[58] Ervin S.M., Li H., Lim L., Roberts L.R., Liang X., Mani S., Redinbo M.R. (2019). Gut Microbial β-Glucuronidases Reactivate Estrogens as Components of the Estrobolome That Reactivate Estrogens. J. Biol. Chem..

[59] Larnder A.H., Manges A.R., Murphy R.A. (2025). The Estrobolome: Estrogen-metabolizing Pathways of the Gut Microbiome and Their Relation to Breast Cancer. Int. J. Cancer.

[60] Bucurica S., Lupanciuc M., Ionita-Radu F., Stefan I., Munteanu A.E., Anghel D., Jinga M., Gaman E.L. (2023). Estrobolome and Hepatocellular Adenomas—Connecting the Dots of the Gut Microbial β-Glucuronidase Pathway as a Metabolic Link. Int. J. Mol. Sci..

[61] Agans R., Rigsbee L., Kenche H., Michail S., Khamis H.J., Paliy O. (2011). Distal Gut Microbiota of Adolescent Children Is Different from That of Adults: Gut Microbiota of Adolescents Differs from That of Adults. FEMS Microbiol. Ecol..

[62] Lephart E.D., Naftolin F. (2022). Estrogen Action and Gut Microbiome Metabolism in Dermal Health. Dermatol. Ther..

[63] Calcaterra V., Rossi V., Massini G., Regalbuto C., Hruby C., Panelli S., Bandi C., Zuccotti G. (2022). Precocious Puberty and Microbiota: The Role of the Sex Hormone–Gut Microbiome Axis. Front. Endocrinol..

[64] Huang X., Chen J., Zou H., Huang P., Luo H., Li H., Cai Y., Liu L., Li Y., He X. (2023). Gut Microbiome Combined with Metabolomics Reveals Biomarkers and Pathways in Central Precocious Puberty. J. Transl. Med..

[65] Lange O., Proczko-Stepaniak M., Mika A. (2023). Short-Chain Fatty Acids-A Product of the Microbiome and Its Participation in Two-Way Communication on the Microbiome-Host Mammal Line. Curr. Obes. Rep..

[66] Enck P., Zimmermann K., Rusch K., Schwiertz A., Klosterhalfen S., Frick J.S. (2009). The Effects of Maturation on the Colonic Microflora in Infancy and Childhood. Gastroenterol. Res. Pract..

[67] Fan Y., Pedersen O. (2021). Gut Microbiota in Human Metabolic Health and Disease. Nat. Rev. Microbiol..

[68] Santos-Marcos J.A., Rangel-Zuñiga O.A., Jimenez-Lucena R., Quintana-Navarro G.M., Garcia-Carpintero S., Malagon M.M., Landa B.B., Tena-Sempere M., Perez-Martinez P., Lopez-Miranda J. (2018). Influence of Gender and Menopausal Status on Gut Microbiota. Maturitas.

[69] Sommer F., Bäckhed F. (2013). The Gut Microbiota—Masters of Host Development and Physiology. Nat. Rev. Microbiol..

[70] Yoon K., Kim N. (2021). Roles of Sex Hormones and Gender in the Gut Microbiota. J. Neurogastroenterol. Motil..

[71] Tremaroli V., Bäckhed F. (2012). Functional Interactions between the Gut Microbiota and Host Metabolism. Nature.

[72] Elderman M., Hugenholtz F., Belzer C., Boekschoten M., Van Beek A., De Haan B., Savelkoul H., De Vos P., Faas M. (2018). Sex and Strain Dependent Differences in Mucosal Immunology and Microbiota Composition in Mice. Biol. Sex Differ..

[73] Qi X., Yun C., Pang Y., Qiao J. (2021). The Impact of the Gut Microbiota on the Reproductive and Metabolic Endocrine System. Gut Microbes.

[74] Hopkins M.J., Sharp R., Macfarlane G.T. (2002). Variation in Human Intestinal Microbiota with Age. Dig. Liver Dis..

[75] Rogers I.S., Northstone K., Dunger D.B., Cooper A.R., Ness A.R., Emmett P.M. (2010). Diet throughout Childhood and Age at Menarche in a Contemporary Cohort of British Girls. Public Health Nutr..

[76] Chakraborti C.K. (2015). New-Found Link between Microbiota and Obesity. World J. Gastrointest. Pathophysiol..

[77] Delzenne N.M., Cani P.D. (2011). Interaction Between Obesity and the Gut Microbiota: Relevance in Nutrition. Annu. Rev. Nutr..

[78] Wang M., Zhang Y., Miller D., Rehman N.O., Cheng X., Yeo J.-Y., Joe B., Hill J.W. (2020). Microbial Reconstitution Reverses Early Female Puberty Induced by Maternal High-Fat Diet During Lactation. Endocrinology.

[79] Bao M., Wu R., Li J., Tang R., Song C. (2025). Research Summary, Possible Mechanisms and Perspectives of Gut Microbiota Changes Causing Precocious Puberty. Front. Nutr..

[80] Sun Y., Liu H., Mu C., Liu P., Hao C., Xin Y. (2024). Early Puberty: A Review on Its Role as a Risk Factor for Metabolic and Mental Disorders. Front. Pediatr..

[81] Cussotto S., Sandhu K.V., Dinan T.G., Cryan J.F. (2018). The Neuroendocrinology of the Microbiota-Gut-Brain Axis: A Behavioural Perspective. Front. Neuroendocrinol..

[82] Singh V., Lee G., Son H., Koh H., Kim E.S., Unno T., Shin J.-H. (2023). Butyrate Producers, “The Sentinel of Gut”: Their Intestinal Significance with and beyond Butyrate, and Prospective Use as Microbial Therapeutics. Front. Microbiol..

[83] Zhai S., Qin S., Li L., Zhu L., Zou Z., Wang L. (2019). Dietary Butyrate Suppresses Inflammation through Modulating Gut Microbiota in High-Fat Diet-Fed Mice. FEMS Microbiol. Lett..

[84] Rodríguez Mazariegos J.R., Nam N.N., Bo T., Wang D., Hsu J.-W., Chen Y.-C. (2025). Precocious Puberty and Gut Microbiome: A Systematic Review and Meta-Analysis. Pediatr. Res..

[85] Bo T., Liu M., Tang L., Lv J., Wen J., Wang D. (2022). Effects of High-Fat Diet During Childhood on Precocious Puberty and Gut Microbiota in Mice. Front. Microbiol..

[86] Wu N., Jiang X., Wang Y., Zhang M., Yue M., Chen F., Wu W., Liu Y., Wang Q., Zhang L. (2025). Gut Microbiota Alterations Modulate High-Fat Diet-Induced Precocious Puberty. Microbiol. Spectr..

[87] Houtman T.A., Eckermann H.A., Smidt H., De Weerth C. (2022). Gut Microbiota and BMI throughout Childhood: The Role of Firmicutes, Bacteroidetes, and Short-Chain Fatty Acid Producers. Sci. Rep..

[88] Facchin S., Bertin L., Bonazzi E., Lorenzon G., De Barba C., Barberio B., Zingone F., Maniero D., Scarpa M., Ruffolo C. (2024). Short-Chain Fatty Acids and Human Health: From Metabolic Pathways to Current Therapeutic Implications. Life.

[89] Morrison D.J., Preston T. (2016). Formation of Short Chain Fatty Acids by the Gut Microbiota and Their Impact on Human Metabolism. Gut Microbes.

[90] Vinolo M.A.R., Rodrigues H.G., Nachbar R.T., Curi R. (2011). Regulation of Inflammation by Short Chain Fatty Acids. Nutrients.

[91] Hosseini E., Grootaert C., Verstraete W., Van De Wiele T. (2011). Propionate as a Health-Promoting Microbial Metabolite in the Human Gut. Nutr. Rev..

[92] Lin H.V., Frassetto A., Kowalik E.J., Nawrocki A.R., Lu M.M., Kosinski J.R., Hubert J.A., Szeto D., Yao X., Forrest G. (2012). Butyrate and Propionate Protect against Diet-Induced Obesity and Regulate Gut Hormones via Free Fatty Acid Receptor 3-Independent Mechanisms. PLoS ONE.

[93] Novaira H.J., Ng Y., Wolfe A., Radovick S. (2009). Kisspeptin Increases GnRH mRNA Expression and Secretion in GnRH Secreting Neuronal Cell Lines. Mol. Cell. Endocrinol..

[94] O’Riordan K.J., Collins M.K., Moloney G.M., Knox E.G., Aburto M.R., Fülling C., Morley S.J., Clarke G., Schellekens H., Cryan J.F. (2022). Short Chain Fatty Acids: Microbial Metabolites for Gut-Brain Axis Signalling. Mol. Cell. Endocrinol..

[95] Xiao L., Wang J., Zheng J., Li X., Zhao F. (2021). Deterministic Transition of Enterotypes Shapes the Infant Gut Microbiome at an Early Age. Genome Biol..

[96] Cho K.Y. (2023). Association of Gut Microbiota with Obesity in Children and Adolescents. Clin. Exp. Pediatr..

[97] Pryde S.E., Duncan S.H., Hold G.L., Stewart C.S., Flint H.J. (2002). The Microbiology of Butyrate Formation in the Human Colon. FEMS Microbiol. Lett..

[98] Ríos-Covián D., Ruas-Madiedo P., Margolles A., Gueimonde M., De Los Reyes-Gavilán C.G., Salazar N. (2016). Intestinal Short Chain Fatty Acids and Their Link with Diet and Human Health. Front. Microbiol..

[99] Nakayama J., Watanabe K., Jiang J., Matsuda K., Chao S.-H., Haryono P., La-ongkham O., Sarwoko M.-A., Sujaya I.N., Zhao L. (2015). Diversity in Gut Bacterial Community of School-Age Children in Asia. Sci. Rep..

[100] Parker B.J., Wearsch P.A., Veloo A.C.M., Rodriguez-Palacios A. (2020). The Genus Alistipes: Gut Bacteria With Emerging Implications to Inflammation, Cancer, and Mental Health. Front. Immunol..

[101] Duncan S.H., Belenguer A., Holtrop G., Johnstone A.M., Flint H.J., Lobley G.E. (2007). Reduced Dietary Intake of Carbohydrates by Obese Subjects Results in Decreased Concentrations of Butyrate and Butyrate-Producing Bacteria in Feces. Appl. Environ. Microbiol..

[102] Pérez-Navero J.L., Benítez-Sillero J.D., Gil-Campos M., Guillén-del Castillo M., Tasset I., Túnez I. (2009). Changes in oxidative stress biomarkers induced by puberty. An. Pediatr..

[103] Tett A., Huang K.D., Asnicar F., Fehlner-Peach H., Pasolli E., Karcher N., Armanini F., Manghi P., Bonham K., Zolfo M. (2019). The Prevotella Copri Complex Comprises Four Distinct Clades Underrepresented in Westernized Populations. Cell Host Microbe.

[104] Wang L., Tang J., Wang L., Tan F., Song H., Zhou J., Li F. (2021). Oxidative Stress in Oocyte Aging and Female Reproduction. J. Cell. Physiol..

[105] Liu C., Zhou S., Li Y., Yin X., Li P. (2024). Metabolomic Disorders Caused by an Imbalance in the Gut Microbiota Are Associated with Central Precocious Puberty. Front. Endocrinol..

[106] Sandhu J., Ben-Shlomo Y., Cole T.J., Holly J., Davey Smith G. (2006). The Impact of Childhood Body Mass Index on Timing of Puberty, Adult Stature and Obesity: A Follow-up Study Based on Adolescent Anthropometry Recorded at Christ’s Hospital (1936–1964). Int. J. Obes..

[107] Calcaterra V., Verduci E., Magenes V.C., Pascuzzi M.C., Rossi V., Sangiorgio A., Bosetti A., Zuccotti G., Mameli C. (2021). The Role of Pediatric Nutrition as a Modifiable Risk Factor for Precocious Puberty. Life.

[108] Lakshmanan A.P., Al Zaidan S., Bangarusamy D.K., Al-Shamari S., Elhag W., Terranegra A. (2022). Increased Relative Abundance of Ruminoccocus Is Associated With Reduced Cardiovascular Risk in an Obese Population. Front. Nutr..

[109] Pai A.H.-Y., Wang Y.-W., Lu P.-C., Wu H.-M., Xu J.-L., Huang H.-Y. (2023). Gut Microbiome–Estrobolome Profile in Reproductive-Age Women with Endometriosis. Int. J. Mol. Sci..

[110] Valsamakis G., Arapaki A., Balafoutas D., Charmandari E., Vlahos N.F. (2021). Diet-Induced Hypothalamic Inflammation, Phoenixin, and Subsequent Precocious Puberty. Nutrients.

[111] Kiess W., Hoppmann J., Gesing J., Penke M., Körner A., Kratzsch J., Pfaeffle R. (2016). Puberty–Genes, Environment and Clinical Issues. J. Pediatr. Endocrinol. Metab..

[112] Valdearcos M., Robblee M.M., Benjamin D.I., Nomura D.K., Xu A.W., Koliwad S.K. (2014). Microglia Dictate the Impact of Saturated Fat Consumption on Hypothalamic Inflammation and Neuronal Function. Cell Rep..

[113] Fujioka H., Kakehashi C., Funabashi T., Akema T. (2013). Immunohistochemical Evidence for the Relationship between Microglia and GnRH Neurons in the Preoptic Area of Ovariectomized Rats with and without Steroid Replacement. Endocr. J..

[114] Chen T., Chen C., Wu H., Chen X., Xie R., Wang F., Sun H., Chen L. (2021). Overexpression of *P53* Accelerates Puberty in High-Fat Diet–Fed Mice through *Lin28/Let-7* System. Exp. Biol. Med..

[115] Yosten G.L.C., Lyu R.-M., Hsueh A.J.W., Avsian-Kretchmer O., Chang J.-K., Tullock C.W., Dun S.L., Dun N., Samson W.K. (2013). A Novel Reproductive Peptide, Phoenixin. J. Neuroendocrinol..

[116] Gu Q., Du Q., Xia L., Lu X., Wan X., Shao Y., He J., Wu P. (2024). Mechanistic Insights into EGCG’s Preventive Effects on Obesity-Induced Precocious Puberty through Multi-Omics Analyses. Food Funct..

[117] Rodríguez-Daza M.C., Pulido-Mateos E.C., Lupien-Meilleur J., Guyonnet D., Desjardins Y., Roy D. (2021). Polyphenol-Mediated Gut Microbiota Modulation: Toward Prebiotics and Further. Front. Nutr..

[118] Cowan C.S.M., Richardson R. (2019). Early-life Stress Leads to Sex-dependent Changes in Pubertal Timing in Rats That Are Reversed by a Probiotic Formulation. Dev. Psychobiol..

